# WHAT SHAPES ADULT SONS’ INCENTIVE FOR PARENT CARE IN JAPAN? EFFECTS OF WORK AND FAMILY CIRCUMSTANCES

**DOI:** 10.1093/geroni/igz038.501

**Published:** 2019-11-08

**Authors:** Tomoko Wakui, Ryo Hirayama, Emily M Agree, Ichiro Kai

**Affiliations:** 1 Tokyo Metropolitan Institute of Gerontology, Tokyo, Japan; 2 Johns Hopkins University, Baltimore, Maryland, United States; 3 The University of Tokyo, Tokyo, Japan

## Abstract

In Japan, because of marked sociodemographic shifts such as a growing number of older adults in need of care and fewer siblings in younger generations, adult sons are increasingly likely to be called upon to meet parental care needs while at working age. Using data from 1,183 employed men (M age = 44.6), our aim in this study was to explore work- and family-related factors that affect their incentives for parental caregiving, a potential component of their psychological preparedness for fulfilling care responsibility. Specifically, focusing on these sons’ workplace environment, living arrangements, and the quality of relationship with parents, we sought to identify whether and how these factors influenced their desire to provide different types of care (e.g., domestic help, ADL assistance). Regression analysis revealed differential associations between work- and family-related factors and sons’ desire for parental caregiving depending on type of care and parents’ gender. For their desire to provide domestic help and attend parents when seeing a doctor, live-in sons were more likely to desire to provide care regardless of parents’ gender. Whereas the quality of parent-child relationships was significantly associated with their desire to care for both parents, whether they had a supervisor to talk with about family issues had a significant effect only on that for their mother. On the basis of these findings, we discuss workplace needs for support to help sons continue work when they need to provide care.

